# Expanding Research on Cannabis-Based Medicines for Liver Steatosis: A Low-Risk High-Reward Way Out of the Present Deadlock?

**DOI:** 10.1089/can.2022.0014

**Published:** 2023-02-06

**Authors:** Tangui Barré, Vincenzo Di Marzo, Fabienne Marcellin, Patrizia Burra, Patrizia Carrieri

**Affiliations:** ^1^ Aix Marseille Univ, Inserm, IRD, SESSTIM, Sciences Economiques & Sociales de la Santé & Traitement de l'Information Médicale, ISSPAM, Marseille, France.; ^2^ Istituto di Chimica Biomolecolare, CNR, Pozzuoli, Italy.; ^3^ Endocannabinoid Research Group, Pozzuoli, Italy.; ^4^ Canada Excellence Research Chair on the Microbiome-Endocannabinoidome Axis in Metabolic Health, CRIUCPQ and INAF-Centre NUTRISS, Faculties of Medicine and Agriculture and Food Sciences, Université Laval, Québec, Canada.; ^5^ Department of Surgery, Oncology and Gastroenterology, Padua University Hospital, Padua Italy.

**Keywords:** cannabidiol, cannabinoids, cannabis, diabetes, insulin resistance, liver steatosis, metabolic disorders, NAFLD, NASH

## Abstract

Obesity and nonalcoholic fatty liver disease (NAFLD) constitute global and growing epidemics that result in therapeutic dead ends. There is an urgent need for new and accessible treatments to improve and widen both preventive and curative approaches against NAFLD. The endocannabinoid system (ECS) is recognized as a complex signaling apparatus closely related to metabolic disorders and is a key target for treating NAFLD. Despite a lack of conclusive clinical trials, observational and pre-clinical studies highlight putative benefits of phytocannabinoids on liver steatosis through multiple pathways. Owing to both its safety profile and its diversity of active compounds acting primarily (although not exclusively) on the ECS—and its expanded version, the endocannabinoidome, the *Cannabis* plant should be considered a major prospect in the treatment of NAFLD. However, seizing this opportunity, and intensifying clinical research in this direction, will require overcoming both scientific and nonscientific barriers.

## Introduction

Obesity prevalence in the upcoming years is expected to increase globally and at the European level.^1,2^ This rise is accompanied by a similar trend for associated metabolic disorders such as diabetes, nonalcoholic fatty liver disease (NAFLD), and nonalcoholic steatohepatitis (NASH),^3,4^ which is an inflammatory and fibrotic stage of NAFLD that can lead to cirrhosis, hepatocellular carcinoma, and end-stage liver disease. A quarter of the world's adult population currently suffers from NAFLD.^3^

Insulin resistance plays a key role in the pathogenesis of NAFLD, which however results also from the interplay between diet, gut microbiota dysbiosis, genetic factors, and *de novo* lipogenesis. Lifestyle changes toward healthier diets and increased physical activity continue to be prescribed as first-line treatments. Nevertheless, long-term adherence to healthier behavioral changes remains a great challenge for patients and their providers.^5,6^

Given the current trends, and the fact that new drugs are still under evaluation to control inflammation and progression toward advanced stages of liver fibrosis,^7,8^ there is an urgent need to offer novel and innovative treatment options to prevent and manage NAFLD. There are complex links relating the endocannabinoid system (ECS) and its extended version—the endocannabinoidome—to metabolic and hepatic disorders.

As cannabinoids can modify ECS functioning, in this perspective article, we argue in favor of intensifying research on full-spectrum cannabis and cannabinoids as promising therapeutic options for metabolic disorders and NAFLD.

## The ECS, a Pro-Homeostatic System

The ECS is a signaling network composed of cannabinoid receptors (CB1 and CB2), their ligands (endocannabinoids), and ligand synthesizing and degrading enzymes. Its expanded version, which includes a plethora of lipid mediators with similar metabolic pathways but often different targets, constitutes the endocannabinoidome.^9^

**FIG. 1.**
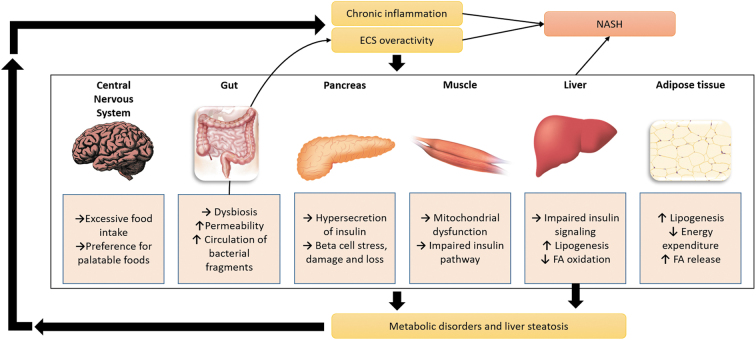
Involvement of the ECS in a vicious cycle leading to liver steatosis and NASH. ECS, endocannabinoid system; NASH, nonalcoholic steatohepatitis.

The ECS and the endocannabinoidome, in particular, are complex systems, expressed through most organs, and involved in many physiological pathways. The ECS is widely involved in food intake and energy homeostasis, including hepatic glucose and lipid homeostasis and, therefore, is connected to metabolic disorders (Fig. 1).^10^ Both CB1 and CB2 are present in liver tissue and mediate a number of biological functions in different types of liver cells. Both receptors, as well as peroxisome proliferator-activated receptors (PPARs), impact lipid metabolism and apparently participate in NAFLD development and its progression to NASH.^11,12^ Specifically, CB1 (and to a lesser extent CB2) antagonism is a strategy to prevent or reduce hepatic steatosis in pre-clinical models.^11^

Importantly, gut dysbiosis, a peculiarity of people with metabolic and associated hepatic disorders, seems to be causally related to alterations of the ECS and the endocannabinoidome, which have been found to regulate gut permeability.^9,13^ Through both direct and indirect effects on the gut microbiome, the endocannabinoidome is also comprehensively involved in the mechanisms of inflammation and oxidative stress, which are key components of obesity and NASH.^9^

In people with obesity^14^ and metabolic disorders (including hepatic steatosis^15,16^), the ECS is overactive (featuring elevated endocannabinoid “tone”), which leads to disruption in homeostasis, contributing to metabolic disorder genesis and/or persistence, thus fuelling a vicious cycle that further disrupts ECS functioning (Fig. 1).^10^

The ECS has been named after the *Cannabis sativa* plant, which produces multiple phytocannabinoids (exogenous cannabinoids coming from plant material) and terpenes that also interact with the ECS and the endocannabinoidome functioning.

The ECS and the endocannabinoidome are sensitive to lifestyle modifications, in particular to the relative intake of n-3 and n-6 polyunsaturated fatty acids, pre- and/or probiotic consumption and physical activity.^9^ The effectiveness of lifestyle interventions on NAFLD/NASH is actually likely mediated in part by the improvement of ECS and endocannabinoidome functioning.

Cannabinoids from *Cannabis* (or “phytocannabinoids”) act on cannabinoid receptors, and several other endocannabinoidome receptors (i.e., PPARα and γ, thermosensitive transient receptor potential channels, and some orphan G-protein coupled receptors, such as GPR6, GPR18, and GPR55).^17^ They also are linked with an impact on gut microbiota composition,^9^ thereby potentially affecting all of the physiopathological conditions that are influenced by intestinal microorganisms.

## The Therapeutic Promises of Cannabinoids for Liver and Metabolic Diseases

### Evidence from observational studies on cannabis use

Observational studies conducted among cannabis users found a significant inverse relationship between cannabis use and body weight, potentially related to a long-lasting downregulation of CB1,^18^ therefore mimicking CB1 blockade. Data regarding cannabis use and liver steatosis tend to display a similar pattern,^19–21^ although null effects have occasionally been reported,^22,23^ as well as isolated negative effects among untreated hepatitis C virus-infected individuals.^24^ In addition, protective effects of cannabis use on the occurrence of insulin resistance and diabetes have also been observed in diverse populations.^25–27^

### Advances in pre-clinical and clinical studies on cannabinoids

**FIG. 2.**
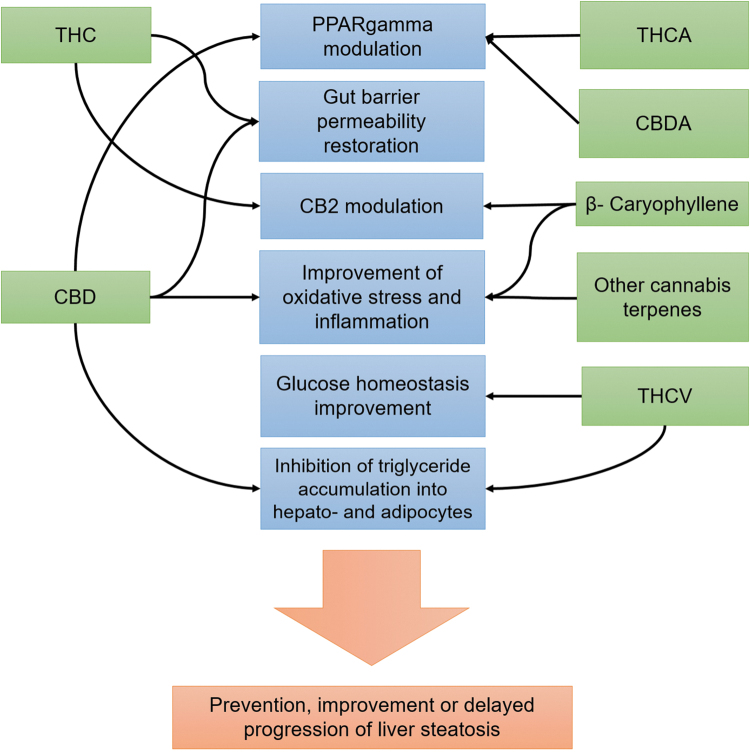
Nonexhaustive pathways by which cannabis chemical components may exert beneficial actions on liver steatosis. Direct modulation of CB1 by CBD and THC is not represented. CB2, cannabinoid receptor 2; CBD, cannabidiol; CBDA, cannabidiolic acid; THC, Δ9-tetrahydrocannabinol; THCA, Δ9-tetrahydrocannabinolic acid; THCV, tetrahydrocannabivarin.

The effects on the liver of the main studied cannabinoids are summarized in Figure 2.

Δ9-tetrahydrocannabinol (THC) and cannabidiol (CBD) are the most abundant and studied phytocannabinoids. As a partial CB1 agonist, THC is likely to be pro-steatotic.^11^ Contrary to THC, CBD, a nonpsychoactive cannabinoid for which a CB1 negative allosteric modulator activity has been suggested, is a promising candidate as an antisteatotic agent,^11^ and has already yielded results in animal and *in vitro* models.^28^ Moreover, as an antioxidant and anti-inflammatory molecule,^29–31^ CBD is likely to protect the liver from progression to NASH.

However, a phase 2 randomized controlled dose-ranging trial comparing 200/400/800 mg CBD per day with placebo in people with liver steatosis (8-week treatment, *n*=25) showed no statistically significant benefit of CBD regarding liver triglyceride levels.^32^ Because 800 mg per day is within the upper range of doses used for CBD and is efficacious for other conditions,^33,34^ this lack of effect is unlikely due to underdosing. Low-dose CBD, alone or in combination with tetrahydrocannabivarin (THCV), also had no impact on liver triglyceride levels in people with diabetes.^35^

THCV, the third most studied phytocannabinoid, acts as a neutral antagonist of CB1 at low doses, and is a competitive antagonist of this receptor only in the presence of its agonists. The clinical benefits of THCV are limited to glucose homeostasis but not liver fat content.^35^

However, promising results on fatty liver were found in *in vitro* and animal models.^28^ PPARα and γ are part of the endocannabinoidome and THC and CBD increase their transcriptional activity, and exert effects that are inhibited by selective antagonists of PPARγ, whereas THCV does not exhibit these properties.^11^ Use of PPARγ agonists is associated with histological improvement in NAFLD.^36^ Finally, expected mechanisms potentially underlying the protection cannabis gives against metabolic disorders include downregulation of CB1 after exposure to THC, and/or direct regulation of appetite, food intake, and food preference through the action of THC and other cannabinoids.^10,18^

Besides the “major” phytocannabinoids, other, still understudied, cannabinoids such as cannabinoid acid precursors in the plant flowers (e.g., tetrahydrocannabinolic acid [THCA] or cannabidiolic acid [CBDA]), may be of interest for NAFLD prevention, due either to their interaction with endocannabinoidome receptors, or, as previously suggested for CBD, their immunomodulatory, anti-inflammatory, or antioxidant actions.^37–39^ Interacting with the intestinal epithelial barrier and microbiota are other pathways that phytocannabinoids may follow to impact NAFLD pathogenesis,^40,41^ in addition to potential psychosocial or behavioral pathways.^42^

A protective effect of rimonabant—a synthetic CB1 antagonist—on obesity-associated hepatic steatosis has been shown in rats,^43^ and on hepatic fat infiltration in severely uncontrolled diabetic rats.^44^ Positive effects have also been obtained in humans.^45,46^ However, rimonabant was quickly withdrawn because of serious psychiatric side effects,^47^ thereby contributing to reduce the interest in using cannabinoids as treatment for metabolic disorders.

To circumvent untoward behavioral effects observed after treatment with brain-penetrant CB1 antagonists, peripherally restricted CB1-targeting drugs have been developed, with positive results on cardiometabolic parameters in obese mice,^48–50^ as well as liver steatosis.^51^ Clinical trials have already started,^52,53^ with drugs deemed ready to move to phase 2 studies,^54^ thus opening the way to a promising new treatment paradigm for obesity-related disorders such as liver steatosis.

### Why we should explore the potential of specific varieties of the *Cannabis* plant as a whole

As valuable as they are, studies on isolated cannabinoids should not make us miss the opportunity that whole-plant cannabis extracts represent. Indeed, the *Cannabis* plant generates a large number of various cannabinoid and noncannabinoids compounds, including fatty acids, terpenoids, and flavonoids, in quantities that vary according to its chemical variety (chemovar or chemotype).^55,56^ Functional interactions between CBD and THC have been highlighted.^57^ More broadly, the pharmacological contributions of minor cannabinoids or noncannabinoid compounds have been highlighted and popularized under the term “entourage effect.”^55^

Through synergistic mechanisms between different cannabis chemical components, “full-spectrum” cannabis extracts may thus have different and potentially superior effects compared with those observed with purified major cannabinoids.^55^ A parallel could be made with the effectiveness of the Mediterranean diet, superior to the one of isolated nutrients.^58^ Interactions between different cannabis compounds may also participate in reducing some side effects or adverse events associated with the treatment with single phytocannabinoids.^55^ Moreover, direct effects of noncannabinoid components found in full-spectrum extracts (e.g., limonene, β-caryophyllene or other terpenes) on metabolic disorders should not be excluded.^59–62^

If these nonpurified extracts are prepared following standardized and reproducible Good Manufacturing Practices, and tested in controlled clinical trials, they might provide better evidence as potential treatment of NAFLD than anecdotal beneficial effects of cannabis use on NAFLD. They may represent an alternative to isolated cannabinoids, as they are expected to partly act through several more biological pathways than pure compounds.

## Prospects for Future Research

The astounding plasticity of the *Cannabis* genome, as well as the identification of the enzymes for the production of the major phytocannabinoids, could soon enable the agricultural production of plants with high levels of desired compounds.^55^ Finding proper synergistic cocktails of active cannabis components is key, and characterization and chemical profiling of strains for a specific medical use should become an important step of the research process toward the development of cannabis-based treatments.

In the same way that fully standardized chemotypes of the *Cannabis* plant have been developed as nabiximols (Sativex^®^) for clinical use against spasticity in multiple sclerosis, it would be possible to develop chemotypes rich in CBD, THCV, and/or β-caryophyllene (a terpene with CB2 agonist action found also in other plants^63^) for the prevention or treatment of NAFLD. However, and importantly, the pharmacological and toxicological characteristics of understudied cannabis components will have to be investigated before they are quantitatively incorporated in such composite medications.

An advantage of full-spectrum (i.e., whole flower) cannabis material or extracts is that they can be produced according to frugal innovation principles (i.e., substantial cost reduction, concentration on core functionalities, and optimization of performance levels^64^). This would facilitate production in limited resource settings, although at the expense of the standardization and reproducibility (and perhaps even the safety) of these treatments.

## Remaining Barriers to Overcome and Fears to Dispel

One of the main barriers of expanded research on medical cannabis is the fear of its psychiatric side effects and risk of dependence, both caused mainly by THC through activation of CB1 receptors. Psychiatric side effects and dependence risks would be minimized in a medical context. Indeed, both are related to long-term and/or intense cannabis use, and in particular to the heavy use of THC-rich cannabis varieties.

The presence of significant proportions of specific cannabinoids such as CBD in cannabis products can attenuate the effects of THC.^57^ Delivering cannabis in a medical context would imply being able to control the quantity and quality of the cannabinoid component profile. Prescription of cannabis medicines after medical consultation would imply checking for risk factors for psychiatric disorders and dependence, drug–drug interactions,^65^ adjusting posology and/or redefining the optimal cannabis-based product according to side effects, tolerance, and liver disease progression.

Safe and effective routes such as smoke-free inhalation or oromucosal administration^66^ should be privileged as opposed to smoke-based routes. Moreover, according to the route of administration adopted, the dose–response has to be carefully explored, as it may narrow the therapeutic window of phytocannabinoids.^67^

Before the cannabinoid-related field of research is fully explored, several barriers need to be overcome, such as legal restrictions and supply barriers.^68^ Furthermore, as seen among health care providers,^69^ the stigma associated with cannabis use is present and likely to affect all people involved in research programs, from funding institutions to laboratory directors. These nonscientific barriers must not prevent us from seizing the opportunity to explore novel potential treatments to alleviate the growing prevalence of metabolic disorders and their hepatic complications.

## Conclusions: Can Cannabis Provide the Real “Cure”?

Cannabis and cannabinoids must follow the same rigorous, vigilant, and objective evaluation processes as any other promising candidate treatment for hepatic steatosis. The current knowledge on the impact of nutrition and physical activity on the ECS^9,70,71^ encourages combining cannabis-based medicines and lifestyle changes into comprehensive patient-centered approaches to ensure improvements not only on clinical outcomes but also in quality of life in people at risk of advanced stages of liver disease.

## Abbreviations Used

CBD: cannabidiol
ECS: endocannabinoid system
CBDA: cannabidiolic acid
NAFLD: nonalcoholic fatty liver disease
NASH: nonalcoholic steatohepatitis
PPARs: peroxisome proliferator-activated receptors
THCA: tetrahydrocannabinolic acid
THC: Δ9-tetrahydrocannabinol
THCV: tetrahydrocannabivarin
